# Exploring the Relationship Between Neighborhood-Built Environment and Elderly Health: A Research Based on Heterogeneity of Age and Gender Groups in Beijing

**DOI:** 10.3389/fpubh.2022.882361

**Published:** 2022-05-31

**Authors:** Jingwei Li, Li Tian, Wei Ouyang

**Affiliations:** ^1^School of Architecture and Design, Beijing Jiaotong University, Beijing, China; ^2^School of Architecture, Tsinghua University, Beijing, China; ^3^School of Public Administration and Policy, Renmin University of China, Beijing, China

**Keywords:** neighborhood built environment, elderly health, multilevel structural equation model, age, gender

## Abstract

**Background:**

The built environment quality of neighborhoods has a significant impact on the health of the elderly. Although there has been a wealth of studies on low-density Western cities, research on the impacts of built environment on elderly health in high-density Chinese cities is far from sufficient. The pathways by which the built environment affects elderly health remain to be observed, particularly whether such pathways vary for different ages and genders.

**Methods:**

Based on the data of the “Fourth Survey on the Living Conditions of the Elderly in China” in 2015, a sample survey of 3,360 older adults, aged 60 years and over, in Beijing was conducted. We first explored the built environment factors that affect elderly health with a multilevel regression model. We then adopted a multilevel structural equation model (MSEM) to reveal the mediating effect of health activities. Moreover, a stratified analysis was applied to explore the impact of age and gender heterogeneity on the relationship between built environment and elderly health.

**Results:**

(1) Neighborhood-built environment measured within a 500 m buffer area had a higher correlation with elderly health when compared with other areas. (2) Physical activity and social interaction played a mediating role in the correlation between the built environment and elderly health. Even if the interference of residential self-selection was controlled within the subgroups, majority of the built environment elements had significant impacts on elderly health. (3) The impacts of built environment variables on elderly health vary among different age and gender groups. Population density mainly promoted elderly health through health activities in the middle-aged (aged 70–79) group and high-aged (aged 80+) group, and shorter distance to transit stations affects health by promoting the social interaction among high-aged males (aged 80+) group compared to with other groups, and high-quality built environment significantly promotes physical activity to alleviate loneliness only in elderly females.

**Conclusion:**

To effectively boost elderly health by improving the quality of the built environment, adequate considerations should also be given to the differentiated demands of adults of different age and gender groups, precise health behavior interventions should be provided, and the construction of personalized aging-friendly and livable spatial environments should be emphasized to realize healthy and active aging.

## Introduction

At present, the world has been experiencing an accelerated aging process, and the global elderly population is expected to increase from 900 million in 2015 to 2.1 billion by 2050, with 30% of the increase coming from the rise in the number of the elderly in China (1). How to deal with the health challenges brought by population aging is a serious challenge facing the Chinese society. Beijing is one of the mega-cities within China which faces the highest rate of aging. By the end of 2020, the total number of the registered elderly population in Beijing was 3.786 million, accounting for 27% of the total registered population. This means, for the registered population, one out of every four Beijing residents is aged 60 or above (2).

Physical performance and social adaptability decrease when one ages, making older adults a high-risk group accompanied by more illnesses and disabilities (3). Several studies have shown that the built environment plays a significant role in health, in addition to individual genetics, socioeconomics, behavioral habits, and medical care (4, 5). Due to functional and mental decline and consequent reductions in mobility and social contacts, the elderly tend to spend more time at home and in their neighborhoods. The health of this population is therefore more vulnerable to barriers in their surrounding environment compared with other age groups (6). In 2019, the World Health Organization (WHO) launched the “Decade of Action on Healthy Aging 2020–2030.” This plan attempts to improve the quality of the environment to actively promote healthy aging. The WHO emphasizes the positive significance of the built environment for elderly health and the construction of urban and neighborhood-built environments that provide environmental support for older adults to be able to live, work, play, and age better.

Recently, growing studies have confirmed the impact of neighborhood-built environments on elderly health (7, 8). Most of the studies have been conducted in Western cities. However, these results may not be applicable to high-density cities of China due to different contexts (9). Meanwhile, measuring built environments in neighborhoods of different buffers and exploring their relationship with health may lead to unstable results, which was termed, the “Uncertain Geographic Context Problem (UGCoP)” by Kwan (10). Therefore, it is important to explore how the extent of neighborhood buffers affect elderly health. Moreover, a limited number of studies have found that age and gender disparities affect the relationship between neighborhood-built environment and elderly health. However, the pathways leading to such differences are currently unclear.

Using data from 3,360 older residents in Beijing, this study aims to address the gaps in the research by better understanding the relationships between the neighborhood-built environment and elderly health using a multilevel structural equation modeling (MSEM) approach. More specifically, we aim to examine the following: (1) the effects of the built environment on various health outcomes among older adults, focusing on the impact of buffer delineation on the analysis results; (2) the pathway of the built environment on elderly health through the mediator of health activities, and verifying the stability of the model after controlling for self-selection; (3) whether the pathways through which the influences of the built environment on elderly health differ across age and gender groups.

This study is structured into six sections. Section Literature Review reviews the literature on the effects of the built environment on elderly health. This is followed by section Materials and Methods where the data and method are presented. Section Results details the estimated results. Section Discussion discusses the main findings and the limitations while section Conclusion concludes the study with implications for planning practices.

## Literature Review

Neighborhoods are typically considered to be relatively small areas surrounding a residence and measured by administrative boundaries or buffer zones (11). One current challenge is to determine the appropriate scale for defining neighborhoods (12). Without a fair justification, the choice of neighborhood-scale may lead to unstable results for the relationship between the built environment and health. Most previous studies have used a single census tract as a proxy for the neighborhood. However, for high-density cities, neighborhood interactions are more frequent, and using a single geographic scale to explore neighborhood effects seems too simplified (13).

The built environment refers to “the man-made space, in which people live, work, and recreate on a day-to-day basis” (14). Neighborhoods are the primary daily living and activity spaces for older adults, and many studies have shown the correlation between the quality of their built environment and the health outcomes of the elderly (15, 16). The attributes of the neighborhood-built environment are generally divided into density, diversity, design, destination accessibility, and distance to transit, namely the “5D” framework (17, 18). It is found that increasing neighborhood density reduces the risk of chronic diseases because residents can engage in more physical activity (19), while others have reported negative effects, suggesting that high-density environments may reduce outdoor space and increase stress, which is harmful to physical health (20). Inoue et al. (21) found that increasing land-use diversity is correlated with higher levels of physical and mental health because residents living in these areas can meet multiple needs by having easy access to resources. Most existing research confirms that street connectivity is influential to residents' health-related outcomes by improving the walking ability in older adults (22). Regarding destination accessibility, studies have provided more consistent results, with the higher provision of such services (i.e., recreational, health, and shopping services) predicting better health status of older adults (23). Distance to transit is another element that affects elderly health, as transit is widely viewed as an opportunity for physical activity (24).

The “5D” framework focuses on the flat dimension of the built environment, and some studies have focused on the characteristics of the three-dimensional (3D) perspective. The elements of the 3D perspective-built environment are generally derived from streetscape images, including indicators, such as architecture, greenery, and sky visibility (25, 26), but limited studies have attempted to explore the association between 3D perspective-built environment and health. More attention has been paid to green visibility, and a study in the Netherlands found a positive correlation between green visibility on streets and their attractiveness to older adults (27). People are more likely to stay on the street and engage in activities when they have a positive perception of the environment.

Another challenging task is identifying causal pathways between neighborhood-built environment and population health. Physical activity provides a nexus, specifically, built environments can either facilitate or discourage physical activity that influences health and wellbeing (28). Neighborhoods with higher density, more mixed land use, and better street network connectivity may increase the possibility of walking for transportation among older adults (29). Proximity to public transport facilities has also been shown to be a significant source of physical activity (30). Physical activity can decrease the risk of chronic disease, depression, some types of cancer, and all causes of mortality (31). Another pathway is social interaction. People need social interaction to improve their physical and mental health (28). Built environments can affect social outcomes by providing public and social spaces, such as easy access to public transportation and higher walkability (32, 33). Social interaction includes many forms, including conversations, shared activities, and so on. Research has shown that social interaction can affect behavioral, psychological, and physiological health by influencing healthy behaviors, providing social support, and increasing access opportunities *via* social networks (34, 35).

Due to the effects of aging on physical function and gender differences in environmental perception and family roles, the pathways of built environment effects on elderly health may differ by age and gender (36). Some studies have found that social interaction presents a stronger effect on elderly health than walking activities as aging increases (37). Other studies suggest that the built environment may play a greater role in female health, as females spend more time in the neighborhood compared to males (38). However, there are still limited studies focusing on the difference between the pathways.

In terms of research methods, regression analysis is widely used, such as the multi-level model. However, few studies have been able to prove the causal relationship between built environment and health. Some research has attempted to explore the mediating variables by using structural equations or path analysis, but they ignore the nested structure of the data, which reduces the accuracy of the results (35). Self-selection is also an important factor to be considered when exploring the impacts of the built environment on health. Few studies have conducted an in-depth exploration of pathways by including variables, such as travel preference and lifestyle for residence choices in the regressions (39). In most studies, the emphasis has been on one dimension of health (e.g., disease, mental health) or general health (e.g., self-rated health). However, few studies to date, address the different dimensions of health at the same time.

In general, the relationship between neighborhood-built environment and elderly health is complex. Current research has not reached a consensus on how to define the appropriate scale of the neighborhood. More importantly, existing studies have not conducted an analysis of the pathways between the built environment and elderly health based on the heterogeneity of different groups, for instance, different ages and gender. Therefore, more scientific evidence is needed to understand the relationship between neighborhood characteristics, health activities, and elderly health.

## Materials and Methods

### Data and Sample

The research data consisted of two categories. The first is the individual-level sociodemographic and health-related data of the elderly, which is collected from the “Fourth Survey on the Living Conditions of the Elderly in China” in 2015. The survey was organized by the China Committee on Aging and adopted a multi-stage and multi-level sampling method proportional to the population scale, targeting older adults aged 60 and above, with samples covering 31 provincial-level administrative regions (excluding Hong Kong, Macao, and Taiwan), with a total sampling ratio about 1‰. Each selected respondent was numbered by sampling. The trained surveyors went directly to the households and conducted face-to-face interviews with the respondents to ensure that the respondents fully understood the survey content and the surveyors filled in the questionnaire according to the respondents' answers. The study selected the representative Chinese megacity, Beijing (the capital), which has a hot and rainy summer and a cold and dry winter, with a total area of 16,410.54 km^2^ and a resident population of 21.886 million at the end of 2021. In this study, 112 neighborhoods in Beijing were selected as samples, covering seven administrative districts: Haidian, Dongcheng, Xicheng, Chaoyang, Fengtai, Fangshan, and Shunyi. Each neighborhood had a sample of 30 older adults, and the total number of samples was 3,360. We removed the invalid samples with information loss, finally preserving 2,778 valid samples. The second category is built environment data at the neighborhood level. The data mainly comes from point of interest (POI) data, the 2014 land-use status map of Beijing, and Baidu Street View images.

### Measures

#### Dependent Variables

In this study, we evaluated the elderly health status from two aspects: physical health and mental health. Regarding physical health, chronic disease was selected as a surrogate. Respondents were asked to answer whether they suffered from the following eight categories of chronic diseases, including hypertension, diabetes, cardiovascular diseases (e.g., coronary heart disease, angina pectoris, etc.), gastritis, arthritis, chronic respiratory diseases (e.g., lung diseases, asthma, etc.), malignant tumors, and reproductive system diseases. The number of chronic diseases was taken as a count variable, with higher values representing a higher risk of chronic disease in older adults (40).

Regarding mental health, loneliness and subjective wellbeing were selected as variables. In terms of loneliness, respondents were asked: “Do you feel lonely?” and the responses included “never lonely,” (1) “sometimes lonely,” (2) and “often lonely” (3). Loneliness can lead to fear, sadness, and anxiety in older adults, which can affect sleep and endanger mental health (41). For subjective wellbeing, respondents were asked: “Do you feel happy in general?” allowing for responses on a 5-point Likert scale, ranging from 1 (very unhappy) to 5 (very happy). Subjective wellbeing is a comprehensive assessment of the quality of life that emphasizes an individual's overall feelings about their life situation (42). Higher values on both scales represent higher feelings of loneliness and subjective wellbeing.

To measure self-rated health, respondents were asked: “How would you describe your present health status?” allowing for responses on a 5-point Likert scale, ranging from 1 (very poor) to 5 (very good), with higher values indicating better self-rated health. This scale is commonly used as a measure of general health in population surveys. The measurements also have proven to be stable and effective predictors of objective health, such as mortality or morbidity, and remain valid after controlling for medical, psychological, social, and health-related behaviors (43, 44).

#### Independent Variables

The measurement of neighborhood-built environment is constructed based on the “5D” framework (17), which mainly focuses on flat dimensional indicators. However, the neighborhood environment of people's daily life is three-dimensional in space, and the human body's perception of the built environment in a 3D perspective can also have an important impact on its behavioral activities. Therefore, this research adds elements of the built environment perceived from a 3D perspective to more visually quantify the perception of the environment based on a human scale.

Density is represented by population density. The land-use mix was calculated for diversity, based on the concept of information entropy, and the entropy index was constructed by dividing the land-use into five categories: residential, commercial retail and office, entertainment, institutional, and other land uses (45, 46). Design refers to street-network connectivity, including road density and intersection density. Destination accessibility was measured by the density of various types of facilities. We divide the facilities into four categories according to the daily use of the elderly, including commercial, recreational, leisure, and medical. Distance to transit was defined as the density of bus stops and subway stations. Regarding 3D environmental perception, we measured the visual rate of greenery, sky, and buildings. The built environment elements in the street-view images were extracted by deep learning and processed by fisheye image projection, and the calculation method was based on the Johnson and Watson equation (47). Table 1 describes the definition of neighborhood-built environment variables.

**Table 1:** Built environment measures.

**Dimensions**	**Measure**	**Definition**
Density	Population density	Count of resident population/area of neighborhood administration (10,000 people/km^2^)
Diversity	Land use mix	${\frac{-\sum_{k=1}^{k}p_{k,i}\text{In(}p_{k,i})}{\text{In}(K,i)}}^{①}$
Design	Road network density	Length of road/area of buffer (km/km^2^)
	Intersection density	Number of intersections with 3 or above/area of buffer (pcs/km^2^)
Destination accessibility	Commercial facility density	Number of food, shopping and finance facilities/area of buffer (pcs/km^2^)
	Recreational facility density	Number of culture and entertainment facilities/area of buffer (pcs/km^2^)
	Leisure facility density	Number of parks, squares, gymnasiums and gyms/area of buffer (pcs/km^2^)
	Medical facility density	Number of pharmacies, neighborhood hospitals and general hospitals/area of buffer (pcs/km^2^)
Distance to transit	Bus stop density	Number of bus stops/area of buffer (pcs/km^2^)
	Subway station density	Number of subway stations/area of buffer (pcs/km^2^)
Three-dimensional perception	Green visibility rate	
	Sky visibility rate	${\frac{1}{2\pi }\sin\frac{\pi }{2n}\sum_{i=1}^{n}\sin\left[\frac{\pi \left(2n-1\right)}{2n}\right]a_{i}}^{②}$
	Building visibility rate	

*① K (number of land-use types within buffer i), ^*p_k,i_*^ (proportion of land-use types of type k within buffer i) (48). The formula for land-use mix presented ranges from 0 to 1, and a high score indicates high heterogeneity of land use*.

*② In the fisheye image, n is the total number of divided concentric rings, i is the ring index, which represents the angular width of greenery, sky, and buildings within the ith ring*.

By considering the Uncertain Geographic Context Problem, we created five scales of buffers, including the administrative boundary of the neighborhood, 300, 500, 800, and 1,000 m buffers (10, 49). The selection was based on the concept of the “living circle” in the “Code for Planning and Design on Urban residential Areas” in China, combined with the walking speed of the elderly (0.8 m/s) (50). Based on the administrative boundary, the buffer was constructed with a radius of 5, 10, and 15 min reachable distance by walking for the elderly. This spatial scale is significantly smaller than that of the young and middle-aged people, which is also the premise of our study to measure neighborhood-built environment characteristics for the elderly group. In addition, the maximum radius of public service facilities proposed in the “Code for Planning and Design on Urban residential Areas” is 1,000 m, which is about 20 min walking distance for the elderly, so the 1,000 m buffer was also included in our analysis.

#### Mediating Variables

As mentioned above, physical activity and social interaction are selected as two key mediators.

Physical activity is referred to as a leisure physical activity in which the elderly participated, including walking, jogging, swimming, and other physical exercise behaviors. It was evaluated according to the number of times the respondent participated in physical activity during the previous week: 1 point if they had not exercised in the last week, 2 points if they exercised less than once, 3 points if they exercised once or twice, 4 points if they exercised three to five times, and 5 points if they exercised six times or more. The higher score represents the higher physical activity level of the elderly.

Social interaction was defined as various activities, including public welfare, cultural, recreational, and mutual assistance activities (51). Public welfare activities included maintaining neighborhood social security, maintaining the neighborhood environment, and participating in cultural and scientific promotion activities. Cultural activities included participating in senior citizen associations and universities. Recreational activities included group dancing (square dancing/YangYang song), ball sports (gateball/table tennis/badminton, etc.), and chess and card games (mahjong/card games/chess, etc.). Mutual assistance activities included helping to mediate neighborhood disputes, helping neighbors, and caring for and educating the next generation (excluding grandchildren). If the elderly often participated in one of the above types of social interaction during the past 12 months, the score was set to “1,” and if they did not participate in a certain type of activity, the score was set to “0.” The sum of the scores of the four types of activities was used as the evaluation score of their social interaction, and the score was set in the range of 1 to 4, with higher values indicating a higher level of social interaction.

#### Control Variables

The sociodemographic characteristics of individuals are key factors influencing the elderly health, and are selected as control variables (9, 36), including gender (male, female), age (60–69, 70–79, 80+), residence status (number of cohabitants), education attainment (no formal education, primary school, middle school, high school, postsecondary school, college/university), annual household income (0–1, 1–3, 3–5, 5–10, 10+), and medical examination habits (yes, none).

### Statistical Analysis

Multilevel Regression Model and SEM were applied in this study. Multilevel modeling has been widely used in studies of neighborhood environments and health outcomes (52) because it allows for the simultaneous estimation of the effects of individual-level and group-level factors. The SEM has some advantages in dealing with the quantitative study of the interaction between multiple variables. The data had a clustered structure with older adults nested within the neighborhood, that is, the older adults living in the same neighborhood share the same environmental exposure. Moreover, there are complex interactions between the built environment, health activities, and health outcomes, so MSEM is more appropriate for analysis. Before constructing the model, six dimensions of variables, such as density, diversity, design, destination accessibility, distance to transit, and 3D were constructed based on 13 built environment indicators. Since the built environment indicators are all derived from objective measurement data rather than scale data, the traditional method of constructing latent variables is no longer applicable. Based on the relevant literature, this study used logistic regression to construct latent variables (53). The three steps of the analysis are as follows:

The first step was to construct a multi-level multiple regression model. Based on the five buffer scales of neighborhood administrative boundaries, 300, 500, 800, and 1,000 m buffers were used to measure the built environment characteristics. We then identified the built environment factors affecting the elderly health based on the results and defined at which scale of the geographic unit the relationship between the built environment and elderly health is more appropriate.

The second step was to construct the MSEM. Mediating variables were incorporated to examine the pathways of health activities. In addition, the stability of the model was verified by avoiding residential self-selection. The specific method was to screen out the elderly living in work-unit compounds and affordable housing neighborhoods as subgroups, including 48 neighborhoods with 1,222 elderly people, accounting for 44% of the total sample.

The third step was to construct a heterogeneity analysis model. Based on the stratified analysis, we explored whether the pathways of neighborhood-built environment and elderly health differed by age and gender. For the age heterogeneity model, the study sample was divided into three groups: low-aged group (persons aged 60–69), middle-aged group (persons aged 70–79), and high-aged group (persons aged 80+). For the gender heterogeneity model, the study sample was divided into two groups: elderly males and elderly females. The study combined Fisher's combination test for heterogeneity effect and set the number of sampling times to 1,000 based on bootstrap.

The analysis was conducted using Mplus7.4.

## Results

### Descriptive Statistics

Descriptive statistics for the study population are given in Table 2. Over half of the respondents were female (52.99%), or low-aged elderly (56.30%). The majority was presented as non-living alone, educated at primary level or above (95.21%), had an annual household income of more than 50,000 RMB (74.37%), and had the habit of undergoing routine medical exams (65.87%). Overall, the participants exercised three to five times on average per week and they participated in at least one type of social activity on a regular basis. Less than half (44.67%) of the older adults had a good self-rated health status and the majority (80.63%) suffered from at least one chronic disease. For mental health, most older adults never felt alone (83.3%), and believed they were relatively happy (79.91%).

**Table 2:** Descriptive statistics of the study population (*N* = 2,278).

**Variables**	**Mean (SD)/*N* (%)**
**Socio-demographic variables**	
Gender	
Male	1,306 (47.01%)
Female	1,472 (52.99%)
Age	
60–69	1,564 (56.30%)
70–79	815 (29.34%)
80+	399 (14.36%)
Number of family members living together	2.72 (1.25)
**Educational attainment**	
No formal education	133 (4.79%)
Primary school	520 (18.72%)
Middle school	927 (33.37%)
High school	529 (19.04%)
Postsecondary school	317 (11.41%)
College/university	352 (12.67%)
**Total annual household income (ten thousands RMB)**	
<1	69 (2.48%)
1–3	231 (8.32%)
3–5	412 (14.83%)
5–10	1,089 (39.20%)
>10	977 (35.17%)
**Medical examination habits**	
Yes	1,830 (65.87%)
No	948 (34.13%)
**Healthy activities**	
Physical activity	4.02 (1.35)
Social interaction	1.03 (1.06)
**Health outcome**	
Self-rated health	3.44 (0.77)
Chronic disease	1.67 (1.29)
Loneliness	1.19 (0.45)
Subjective wellbeing	4.03 (0.72)

Descriptive statistics of neighborhood characteristics are given in Table 3. The average land-use mix tended to increase as the buffer scale increased. The density of road and bus stops was similar in all five buffer scales, while the density of intersections (21.72) and subway stations (0.18) was the lowest within the administrative boundaries. The density of commercial, recreational, and medical facilities decreased as the buffer scale increased, but the density of leisure facilities did not show this pattern. Greenery and sky visibility rates were similar in all five buffer scales while building visibility rates were the highest within the administrative boundaries (0.39).

**Table 3:** Descriptive statistics for neighborhood characteristics.

**Variables**	**Mean (SD)**				
	**Administrative boundaries**	**300 m buffer**	**500 m buffer**	**800 m buffer**	**1,000 m buffer**
Population density	2.75 (2.49)				
Land use mix	0.57 (0.20)	0.7 (0.20)	0.72 (0.20)	0.74 (0.20)	0.75 (0.20)
Road network density	5.44 (3.35)	5.50 (2.71)	5.46 (2.58)	5.46 (2.52)	5.51 (2.49)
Intersection density	21.72 (34.02)	28.94 (25.32)	28.03 (22.67)	28.36 (22.38)	28.31 (22.08)
Commercial facility density	32.01 (32.32)	30.72 (22.36)	29.62 (20.31)	29.81 (19.82)	29.23 (19.48)
Recreational facility density	3.81 (6.52)	3.31 (3.47)	3.25 (2.80)	3.12 (2.52)	3.05 (2.45)
Leisure facility density	2.60 (5.10)	2.64 (3.11)	2.60 (2.74)	2.73 (2.41)	2.68 (2.19)
Medical facility density	7.13 (8.98)	6.42 (4.54)	6.33 (4.20)	6.13 (3.77)	5.85 (3.50)
Bus stop density	4.09 (103.58)	4.40 (2.89)	4.12 (2.27)	4.16 (2.29)	4.17 (2.21)
Subway station density	0.18 (0.89)	0.47 (0.60)	0.44 (0.38)	0.40 (0.32)	0.40 (0.31)
Green visibility rate	0.50 (0.21)	0.56 (0.15)	0.56 (0.15)	0.57 (0.14)	0.57 (0.14)
Sky visibility rate	0.11 (0.08)	0.12 (0.07)	0.12 (0.06)	0.12 (0.05)	0.11 (0.05)
Building visibility rate	0.39 (0.20)	0.33 (0.12)	0.33 (0.12)	0.32 (0.12)	0.31 (0.12)

### Multilevel Regression Analysis

The results of the multilevel regression models of the relationship between the built environment at the five buffer scales and elderly health are summarized in Table 4. We found that the correlation between built environment factors and elderly health varied by geographic scale. The population density was positively correlated with chronic diseases and negatively correlated with subjective wellbeing. Land-use mix was positively correlated with self-rated health, which was found only in the 500 m buffer. Street connectivity had a positive effect on self-rated health and a negative effect on chronic diseases at 300 and 500 m buffers, while it had a positive effect on the subjective wellbeing only within the administrative boundaries. Destination accessibility was negatively correlated with loneliness (all five buffer scales) and positively correlated with subjective wellbeing (except for the 800 m buffer). Distance to transit was positively associated with self-rated health and negatively associated with chronic disease (only in the 1,000 m buffer), while it was positively correlated with subjective wellbeing (only in the 500 m buffer). The 3-D environmental perception had a negative effect on loneliness in the 500 m buffer, and significantly associated with all health outcomes in the 800 m buffer. In general, compared with other buffer scales, there are more built environment elements in the 500 m buffer that were significantly associated with health outcomes, which could better explain the relationship between the built environment and elderly health.

**Table 4:** Statistical results of multilevel regression models for multi-buffer-built environment and elderly health.

	**Administrative boundaries**	**300 m buffer**	**500 m buffer**	**800 m buffer**	**1,000 m buffer**
Self-rated health		Street connectivity (0.057^*^)	Land use mix (0.066^**^), Street connectivity (0.049^*^)	3-d perception (0.169^**^)	Distance to transit (0.136^#^)
Chronic disease		Street connectivity (−0.096^*^)	Population density (0.106^*^), Street connectivity (−0.141^**^)	3-d perception (−0.337^**^)	Distance to transit (−0.239^*^)
Loneliness	Destination accessibility (−0.073^***^)	Destination accessibility (−0.038^*^)	Destination accessibility (−0.049^**^), 3-d perception (−0.275^**^)	Destination accessibility (−0.018^*^), 3-d perception (−0.122^*^)	Destination accessibility (−0.023^*^)
Subjective wellbeing	Street connectivity (0.110^**^), Destination accessibility (0.074^**^)	Destination accessibility (0.056^*^)	Population density (−0.084^*^), Destination accessibility (0.065^**^), Distance to transit (0.104^*^)	3-d perception (0.168^**^)	Destination accessibility (0.049^*^)

*N = 2,778*.

*** *P < 0.001*,

** *P < 0.01*,

* *P < 0.05*,

# *P < 0.1*.

### Multilevel Structural Equation Analysis

Table 5 shows the results of the MSEM of the full sample. Physical activity and social interaction were found to be key pathways through which the neighborhood-built environment influences the elderly health. The following are the effects of the physical activity path-based built environment on elderly health: population density had a negative effect, while land-use mix, distance to transit, and 3D-environmental perception had a positive effect. Higher population density may reduce self-rated health and subjective wellbeing and increase the risk of chronic diseases and loneliness in older adults by reducing the physical activity. Higher land-use mix can reduce the risk of chronic diseases and promote the subjective wellbeing of the elderly by increasing the physical activity. A shorter distance to transit may promote self-rated health and subjective wellbeing and reduce the risk of chronic diseases and loneliness by increasing physical activity. Moreover, better 3-D environmental perception may reduce chronic diseases and loneliness and promote subjective wellbeing by increasing physical activity.

**Table 5:** Statistical results of multilevel structural equation models (MSEMs) for the built environment and elderly health (full sample).

**Variables**	**Physical activity**	**Social interaction**	**Self-rated health**			**Chronic disease**			**Loneliness**			**Subjective wellbeing**		
			**Direct effect**	**Indirect effect**		**Direct effect**	**Indirect effect**		**Direct effect**	**Indirect effect**		**Direct effect**	**Indirect effect**	
				**Physical activity**	**Social interaction**		**Physical activity**	**Social interaction**		**Physical activity**	**Social interaction**		**Physical activity**	**Social interaction**
Population density	−0.154^***^	−0.139^**^	−0.015	−0.006^#^	−0.014^*^	0.052	0.024^**^	0.031^**^	−0.040	0.007^*^	0.005	−0.022	−0.027^***^	−0.036^**^
Land use mix	0.054^#^	0.042	0.060^**^	0.002	0.004	−0.041	−0.008^#^	−0.009	0.024	−0.003	−0.002	−0.023	0.009^#^	0.011
Street connectivity	0.038	0.079^*^	0.040^#^	0.001	0.008^*^	−0.118^**^	−0.006	−0.017^*^	0.022	−0.002	−0.003	−0.017	0.007	0.020^*^
Destination accessibility	0.006	0.038	−0.002	0.000	0.004	0.038^#^	−0.001	−0.008	−0.047^*^	0.000	−0.001	0.056^*^	0.001	0.010
Distance to transit	0.107^***^	0.069^#^	0.025	0.004^#^	0.007^#^	0.070	−0.017^**^	−0.015^#^	−0.001	−0.005^*^	−0.003	0.067	0.019^***^	0.018^#^
3-d perception	0.286^*^	0.184	−0.063	0.010	0.019	−0.036	−0.044^*^	−0.040	−0.254^**^	−0.013^#^	−0.007	−0.149	0.050^*^	0.047

*N = 2,778*.

*** *P < 0.001*,

** *P < 0.01*,

* *P < 0.05*,

# *P < 0.1. All models controlled for individual sociodemographic variables, whose coefficients are not shown in the table due to space constraints. Same below*.

The effects of social interaction path-based built environment on elderly health are as follows: population density had a negative effect, while street connectivity and distance to transit had a positive effect. Higher population density may have the potential to reduce self-rated health and subjective wellbeing and increase the risk of chronic disease among older adults by decreasing social interaction. Higher street connectivity was associated with higher self-rated health and subjective wellbeing and a lower risk of chronic disease among older adults through increased social interaction. Higher proximity to public transportation may promote self-rated health and subjective wellbeing and reduce the risk of chronic disease through increased social interaction.

Table 6 shows the results of the MSEM of the subgroup sample. This study found that the model results after controlling for residential self-selection did not differ significantly from the full sample, with only a few effect pathways changing. For self-rated health, the mediating effect of population density through physical activity was no longer significant, and the mediating effect of population density through social interaction on self-rated health (−0.018) was stronger than that of the full sample (−0.014). The mediating effects of street connectivity through social interaction and distance to transit through physical activity and social interaction on self-rated health were no longer significant. The mediating effects of the neighborhood-built environment through physical activity on chronic disease and subjective wellbeing were similar to that of the full sample. However, the mediating effects of street connectivity and distance to transit through social interaction on chronic diseases were no longer significant, nor were the mediating effects of street connectivity through social interaction on subjective wellbeing significant. For loneliness, the mediating effect of the built environment affecting loneliness through physical activity was consistent with that of the full sample.

**Table 6:** Statistical results of multilevel structural equation models (MSEMs) for the built environment and elderly health (subgroup sample).

**Variables**	**Physical activity**	**Social interaction**	**Self-rated health**			**Chronic disease**			**Loneliness**			**Subjective wellbeing**		
			**Direct effect**	**Indirect effect**		**Direct effect**	**Indirect effect**		**Direct effect**	**Indirect effect**		**Direct effect**	**Indirect effect**	
				**Physical activity**	**Social interaction**		**Physical activity**	**Social interaction**		**Physical activity**	**Social interaction**		**Physical activity**	**Social interaction**
Population density	−0.108^*^	−0.160^*^	0.009	−0.003	−0.018^*^	−0.003	0.024^*^	0.034^*^	−0.035	0.008^*^	0.007	−0.100^*^	−0.020^*^	−0.038^*^
Land use mix	0.075^*^	0.066	0.054^*^	0.002	0.008	−0.115^**^	−0.017^*^	−0.014	0.004	−0.005	−0.003	−0.054	0.014^*^	0.016
Street connectivity	0.011	0.080	0.043	0.000	0.009	−0.129^**^	−0.003	−0.017	0.031	−0.001	−0.004	−0.052	0.002	0.019
Destination accessibility	0.027	0.012	−0.007	0.001	0.001	0.039^#^	−0.006	−0.003	−0.059^*^	−0.002	−0.001	0.046^*^	0.005	0.003
Distance to transit	0.075^*^	0.087^#^	0.039	0.002	0.010	0.103^#^	−0.017^*^	−0.018	−0.004	−0.005^#^	−0.004	0.069	0.014^*^	0.021^#^
3-d perception	0.357^*^	0.223	−0.024	0.010	0.025	−0.047	−0.079^*^	−0.047	−0.278^**^	−0.026^#^	−0.010	−0.088	0.067^*^	0.053

*Notes: N = 2,778*.

** *P < 0.01*,

* *P < 0.05*,

# *P < 0.1*.

### Heterogeneity Analysis

**Figure 1:**
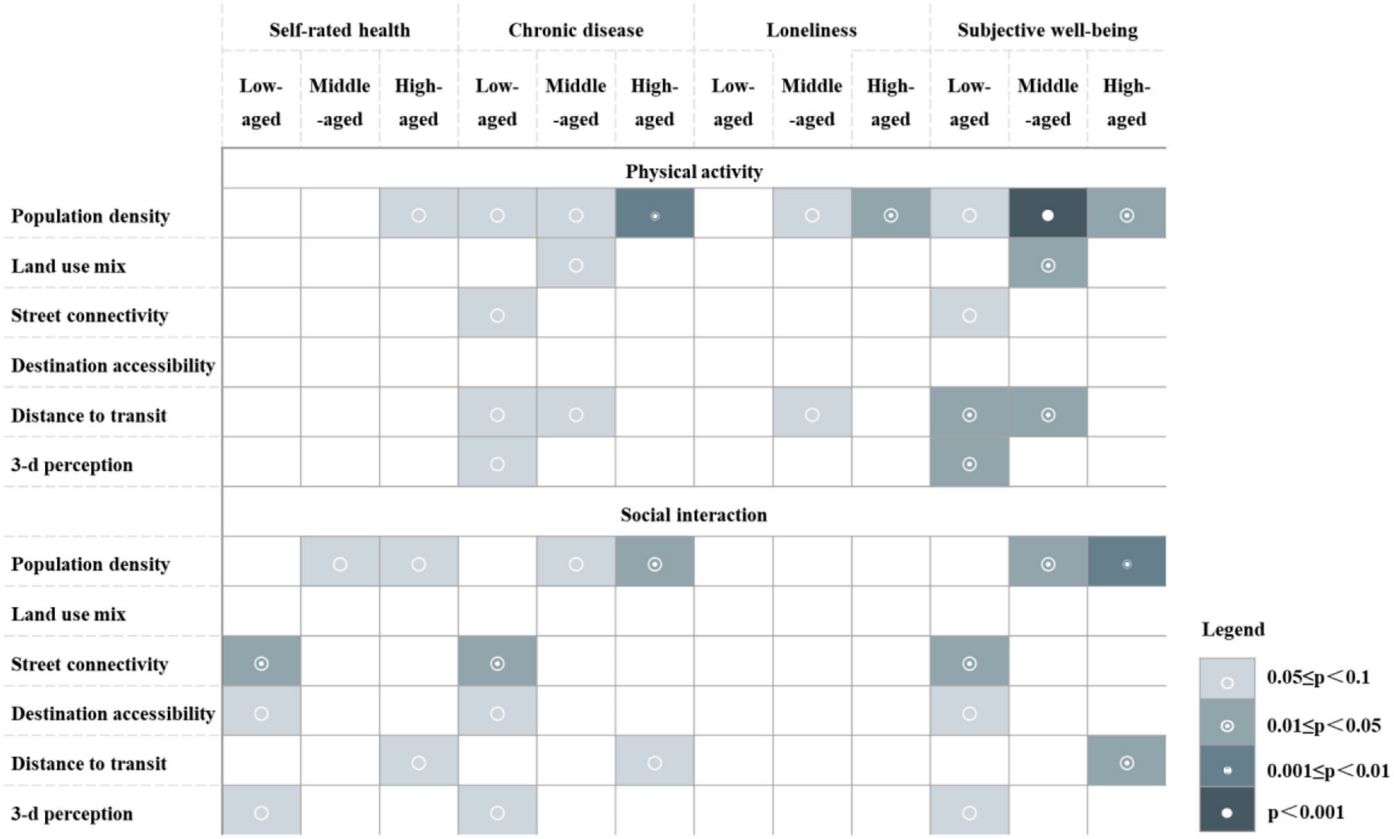
Age heterogeneity of mediating effects of neighborhood-built environment on elderly health.

Figure 1 summarizes the differences in the mediating effects of neighborhood-built environment on elderly health among different age groups. This study found that in the low-aged group (persons aged 60–69), population density, street connectivity, distance to transit, and 3D environmental perception affected chronic disease and subjective wellbeing through physical activity. Street connectivity, destination accessibility, and 3D environmental perception affected self-rated health, chronic disease, and subjective wellbeing through social interaction. In the middle-aged group (persons aged 70–79), population density, land-use mix, and distance to transit were critical for chronic disease and subjective wellbeing by affecting physical activity, but for loneliness, only population density and distance to transit showed a significant impact through physical activity. In the social interaction pathway, only population density played a significant role on self-rated health, chronic disease, and subjective wellbeing. In the high-aged group (persons aged 80+), only population density showed a significant correlation with all health outcomes through physical activity. In the social interaction pathway, both population density and distance to transit showed a significant correlation on self-rated health, chronic disease, and subjective wellbeing.

**Figure 2:**
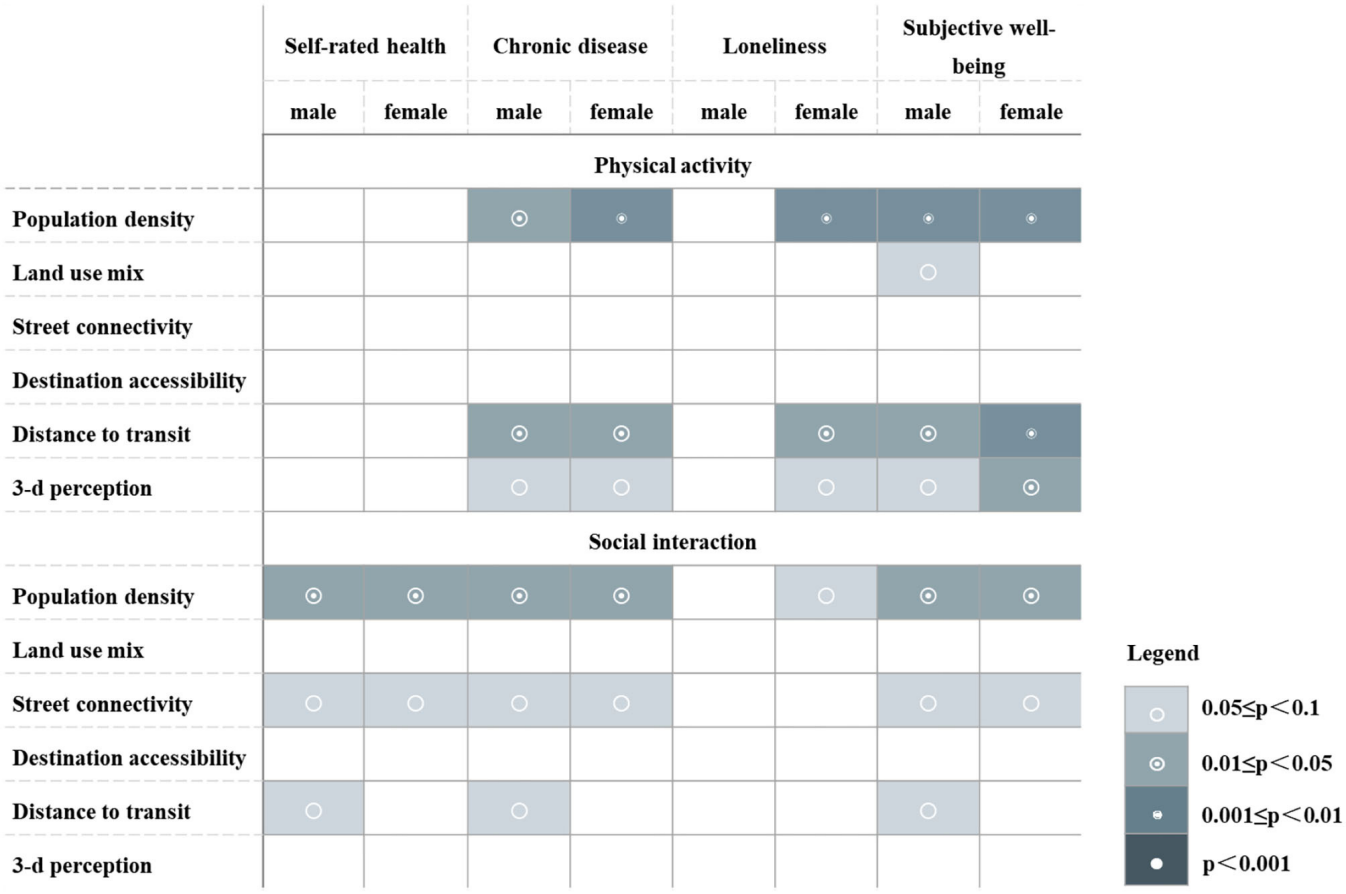
Gender heterogeneity of mediating effects of neighborhood-built environment on elderly health.

Figure 2 summarizes the differences in the mediating effects of neighborhood-built environment on elderly health in different gender groups. The results showed that in the male group, population density, distance to transit, and 3-D environmental perception affected chronic diseases and subjective wellbeing through physical activity, and land-use mix only had a positive effect on subjective wellbeing. In the path of social interaction, population density, street connectivity, and distance to transit showed a significant correlation between self-rated health, chronic diseases, and subjective wellbeing. In the female group, population density, distance to transit, and 3-D environmental perception were found to have a significant effect on chronic disease, loneliness, and subjective wellbeing by physical activity; while population density and street connectivity were associated with self-rated health, chronic disease, and subjective wellbeing through social interaction. However, for loneliness, only population density had a positive effect on it through social interaction.

## Discussion

### Main Findings

This study explored the complex relationship between neighborhood-built environment and elderly health. The results indicate that different characteristics of the built environment played different roles in health outcomes through physical activity and social interaction. There was variability in these pathways among older adults of different age and gender groups. Our main findings are as follows.

### Built Environment Within the 500 m Buffer Has a Higher Correlation With Elderly Health Compared With Other Areas

The choice of neighborhood area definition may dramatically influence the observed relationships between built environment characteristics and elderly health (9). Our research revealed that built environment elements are most significantly related to health outcomes in the 500 m buffer compared with other areas. One possible explanation is that with the increase of age, the physical function of the elderly decreases, resulting in the restriction of their accessibility. The 500 m buffer was selected as the point beyond which daily activity of the elderly begins to decline dramatically and may better capture older adults' daily activity space (54). In addition, neighborhood environmental attributes within the 500 m buffer also appear to be strong predictors of activity for older adults (55). This is consistent with the buffer scale selected in other previous studies conducted in China on the relationship between neighborhood-built environment and elderly health. It suggests that the 500 m buffer can more accurately reflect the built environment characteristics to which older adults are exposed in their daily lives (56). Therefore, the issue of the uncertain geographic context problem cannot be ignored when considering the impact of the neighborhood-built environment on public health. The actual range of daily activities of different groups needs to be considered and the exposure scale of the built environment needs to be defined by multiple comparisons.

### Physical Activity and Social Interaction Are Key Pathways of Neighborhood-Built Environment Effects on Elderly Health

In the path of physical activity, some built environment characteristics could have a positive impact on health through physical activity, which is similar to previous studies (57). Land-use mix, distance to transit, and 3D environmental perception are enablers for outdoor physical activity among older adults. When living in a different land-use area, older adults may have easier access to various facilities and resources, which helps them to achieve higher levels of physical activity (58). Convenient public transport stations can provide various travel routes, which helps encourage the elderly to choose public transportation as a travel mode and increase their physical activity as they walk to transit stops. Previous studies have shown that ~29% of Americans can achieve adequate levels of physical activity only by commuting to and from public transport stations every day (59).

Regarding 3D environmental perception, an increase in the visibility of street greenery implies a more comfortable and pleasant street environment, and older adults are more likely to choose to walk or exercise in such green environments (58). Strong evidence showed that increased physical activity can help promote metabolism, improve body resistance, enhance cardiopulmonary function and flexibility of joints and bones, and reduce the risk of chronic diseases (57). Also, increased interaction with other people during physical activity can be effective in relieving mood and increasing serotonin levels, which can help promote self-rated health and subjective wellbeing and reduce loneliness (60, 61).

It is worth noting, however, that higher population density was negatively associated with health. This is in contrast with many previous studies conducted in Western countries (62, 63). However, the results are in line with many other studies on older adults in Asian cities (64). This is probably due to the fact that older adults are more likely to live in higher-density neighborhoods in Beijing, resulting in a lack of adequate space and places for physical activity. Decreases in physical activity may prolong sedentary time, aggravate the risk of chronic diseases such as obesity, diabetes, and cardiovascular disease, and thus are harmful to health (65).

In the path of social interaction, in line with previous research, our results also suggest that older adults can benefit from high levels of street connectivity and convenient transportation. Higher street connectivity can help encourage older adults to go outdoor for activities and increase their willingness to interact with neighbors (66). Similarly, easy access to public transportation can reduce social exclusion and isolation by increasing physical accessibility (67). As expected, increasing social participation and access to social support for older adults can help generate a positive attitude and a sense of neighborhood attachment and belonging, which is important for maintaining and promoting wellbeing and self-rated health (68). Previous research has also shown that older adults can gain more opportunities to interact with health care providers through social support and discussions with older friends about disease management, which can help them persist in treating chronic diseases (69).

Our study further found that population density had a significant negative impact on health, which is in line with the existing literature (70). In high-density neighborhoods, residents are often exposed to large numbers of strangers, exacerbating the weakening of interpersonal relationships, and reducing social interaction (71). High-density environments may also bring negative psychological feelings, such as psychological stress, anxiety, loss of control, cognitive overload, and violation of personal space (72), reducing the willingness to participate in social activities. Previous studies have shown that inadequate support for social interaction may lead to sleep problems in older adults, resulting in decreased immunity and increased mortality, impairing self-rated health and subjective wellbeing, and increasing the risk of chronic disease (52, 73).

In addition, China's unique housing policy creates an opportunity to control residential self-selection (74). In the model excluding the self-selection samples, some mediating effects are no longer significant, which reflects that individuals who love physical activities and social life are more likely to choose to live in neighborhoods with higher-street connectivity and convenient transportation. However, the subgroups do not differ significantly from the results of the full-sample model in general, and built environment characteristics still play an independent and robust impact. The result may be explained by the fact that the main subjects of the study are local elderly people who have lived in the neighborhood for a longer period and have less self-selection of living location, reducing the possibility of taking the built environment characteristics as the main consideration for housing selection.

### Age and Gender Heterogeneity in the Pathways of Neighborhood-Built Environment Effects on Elderly Health

The relationship between neighborhood-built environment, health activities, and elderly health differed significantly by age group. The role of population density in changing health activities intensifies with increasing age, with significant effects on health outcomes concentrated among older adults in the middle and high age groups. The reason may be that the negative effects of environmental pressure, such as noise and insecurity caused by higher population density on physical activities, social interaction, and other behavior habits gradually appear after enduring them for long periods of time (75). While in the low-aged group, the built environment characteristics that had the most significant positive effects on health included street connectivity, destination accessibility, distance to transit, and 3-D environmental perception, which is consistent with previous research (76). Compared to other age groups, low age elderly has more physical activity and social needs, and higher demands on the quality of their environment (37). For the high-aged group, in addition to population density, no significant relationships were found between other built environment elements and health outcomes through physical activity, indicating that older adults have a weaker perception of the environment conducive to physical activity.

It is interesting to note that the effect of distance to transit on health through physical activity was more significant in the low and middle age groups, but the effect through social interaction was only significant in the high-aged group. The potential explanation might be that compared with the high-aged group, lower age elderly have a wider range of activities, and convenient public transportation facilities have a greater impact on their physical activity. For the high-aged group, the time and intensity of physical activity tended to decline with age, and their range of activity was constrained, resulting in a lower need to rely on public transportation for physical activity. However, previous studies have suggested that the high-aged group elderly have a higher level of need for social interaction and social participation (37) and are more dependent on physical space. As the walking range of the high aged elderly is limited, it is more difficult for them to travel by bike or car for social interaction, and they have a higher demand for public transportation (77).

Moreover, the study found that the relationship between neighborhood-built environment, health activities, and elderly health differed significantly by gender group, but the gender heterogeneity was not as obvious as the age heterogeneity. The effect of distance to transit on health through social interaction was only significant in elderly males. Consistent with the findings from previous studies (78), compared to elderly females who preferred to carry out activities near their neighborhoods, elderly males were more active in social interaction and had a larger spatial range, leading to a higher reliance on public transportation for their outdoor activities. The effect of land use mix on subjective wellbeing through physical activity was only significant in elderly males. The reason might be that elderly males had a wider variety in types of daily exercise activities, such as Taichi, sword dancing, gateball, and other activities. However, the need for elderly females was for facilities more focused on home shopping, which is relatively homogeneous. Therefore, for elderly males, higher land-use mix has a greater impact on physical activity by providing diverse places for physical activity to meet their exercise needs, and improve their subjective wellbeing (79, 80).

Interestingly, the effect on loneliness by improving the built environment was only significant in elderly females but not in elderly males. These characteristics include population density, distance to transit, and 3D environmental perception, which is consistent with previous studies (81). The reason might be that elderly females are more sensitive to the environment and emotions, more dependent on social support and companionship through the external environment, to develop interpersonal relationships in various activities, and to alleviate loneliness.

### Limitations

This study has many limitations. First, the cross-sectional research design is not sufficient for explaining the correlation between neighborhood-built environment and elderly health. More longitudinal data are needed to consider a quasi-natural experiment in order to generate high-quality evidence to provide a more accurate basis for new policy decisions. Second, the study has discussed only two pathways, namely, physical activity and social interaction, and other potential pathways are missing. Further studies should investigate more potential mediation, such as stress exposure, pollution exposure, dietary behavior, and traffic safety, to further deepen the understanding of the relationship between the built environment and health. Last but not the least, this study adopted a static residence-based approach to measure the built environment, which is prone to the problem of uncertain geographic context and average neighborhood effect (82). Future studies should measure the built environment exposure based on individual mobility and incorporate wearable environmental monitoring devices to improve accuracy.

## Conclusion

This study innovatively examined the age and gender differences of the mediation effects between the built environment and elderly health in China. While varying for age and gender, our results indicated that neighborhood-built environment plays an essential role in elderly health through physical activity and social interaction. These findings help enhance our understanding of the elderly health from the perspective of physical environment planning and to realize the health-promoting effects of the neighborhood-built environment. When developing health interventions for the elderly, it is recommended that besides equitable distribution of public services, priority should be given to the spatial layout of various public facilities within the 500 m buffer zone of the elderly residents' daily life. Improvement of the built environment can be done in terms of diversity, design, destination accessibility, distance to transit, and 3-D environmental perception. It should be customized to provide accurate health behavior intervention strategies for the different groups of elderly, according to their behavioral activity needs. In general, further studies on research based on the longitudinal observation of built environment and elderly health are needed to help build a more livable and healthier environment for the aging society.

## Data Availability Statement

The original contributions presented in the study are included in the article/supplementary material, further inquiries can be directed to the corresponding author.

## Author Contributions

Material preparation, data collection, and analysis were performed by JL. The first draft of the manuscript was written by JL. All authors commented on previous versions of the manuscript, contributed to the study conception and design, and have read and approved the final manuscript.

## Funding

This research was sponsored by National Science Foundation of China (51878367), Beijing Outstanding Young Scientist Program (JJWZYJH01201910003010), and the Research Funds for the Major Innovation Platform of Public Health and Disease Control and Prevention, Renmin University of China.

## Conflict of Interest

The authors declare that the research was conducted in the absence of any commercial or financial relationships that could be construed as a potential conflict of interest.

## Publisher's Note

All claims expressed in this article are solely those of the authors and do not necessarily represent those of their affiliated organizations, or those of the publisher, the editors and the reviewers. Any product that may be evaluated in this article, or claim that may be made by its manufacturer, is not guaranteed or endorsed by the publisher.
